# A crowdsourcing database for the copy-number variation of the Spanish population

**DOI:** 10.1186/s40246-023-00466-8

**Published:** 2023-03-09

**Authors:** Daniel López-López, Gema Roldán, Jose L. Fernández-Rueda, Gerrit Bostelmann, Rosario Carmona, Virginia Aquino, Javier Perez-Florido, Francisco Ortuño, Guillermo Pita, Rocío Núñez-Torres, Anna González-Neira, CSVS Crowdsourcing Group, Angel Alonso, Josefa Salgado-Garrido, Sara Pasalodos-Sanchez, Carmen Ayuso, Pablo Minguez, Almudena Avila-Fernandez, Marta Corton, Rafael Artuch, Salud Borrego, Guillermo Antiñolo, Angel Carracedo, Jorge Amigo, Luis Antonio Castaño, Isabel Tejada, Aitor Delmiro, Carmina Espinos, Daniel Grinberg, Encarnación Guillén, Pablo Lapunzina, Jose Antonio Lopez-Escámez, Alvaro Gallego-Martinez, Ramón Martí, Eulalia Rovira, José Mª Millán, Miguel Angel Moreno, Matías Morin, Antonio Moreno-Galdó, Mónica Fernández-Cancio, Beatriz Morte, Victoriano Mulero, Diana García, Virginia Nunes, Francesc Palau, Belén Perez, Luis Pérez Jurado, Rosario Perona, Aurora Pujol, Feliciano Ramos, Esther Lopez, Antonia Ribes, Jordi Rosell, Jordi Surrallés, María Peña-Chilet, Joaquin Dopazo

**Affiliations:** 1 Computational Medicine Platform, Andalusian Public Foundation Progress and Health-FPS, 41013 Seville, Spain; 2 Institute of Biomedicine of Seville, IBiS, University Hospital Virgen del Rocío/CSIC/University of Seville, Seville, Spain; 3 Centro de Investigación Biomédica en Red en Enfermedades Raras (CIBERER), ISCIII, Madrid, Spain; 4 Department of Computer Architecture and Computer Technology, University of Granada, 18071 Granada, Spain; 5 Human Genotyping Unit–CeGen, Spanish National Cancer Research Centre (CNIO), 28029 Madrid, Spain; 6 Navarrabiomed-IdiSNA, Complejo Hospitalario de Navarra, IdiSNA (Navarra Institute for Health Research), Universidad Pública de Navarra (UPNA), Pamplona, Navarre Spain; 7 Department of Genetics, Instituto de Investigación Sanitaria-Fundación Jiménez Díaz University Hospital, Universidad Autónoma de Madrid (IIS-FJD, UAM), Madrid, Spain; 8 Fundación Para la Investigación y Docencia Sant Joan de Deu, Barcelona, Spain; 9 University Hospital Virgen del Rocío, Seville, Spain; 10 Fundación Pública Galega de Medicina Xenómica, SERGAS, IDIS, Santiago de Compostela, Spain; 11 Asociación Instituto de Investigación Sanitaria de Biocruces, Vizcaya, Spain; 12 Hospital Univ. 12 de Octubre, Madrid, Spain; 13 Centro de Investigación Príncipe Felipe, Valencia, Spain; 14 Universidad de Barcelona, Barcelona, Spain; 15 Hospital Virgen de la Arrixaca, Murcia, Spain; 16 Servicio Madrileño de Salud, Madrid, Spain; 17 Department of Genomic Medicine, Centre for Genomics and Oncological Research (GENYO), Pfizer University of Granada, Granada, Spain; 18 Vall d’Hebron Institut de Recerca, Barcelona, Spain; 19 Fundación para la Investigación del Hospital la Fe, Valencia, Spain; 20 Servicio de Genética, Ramón y Cajal Institute of Health Research (IRYCIS) and Biomedical Network Research Centre on Rare Diseases (CIBERER), Madrid, Spain; 21 Vall d’Hebron Institut de Recerca (VHIR), Hospital Universitari Vall d’Hebron, Barcelona, Spain; 22 Undiagnosed Rare Diseases Programme (ENoD), Center for Biomedical Research on Rare Diseases (CIBERER), ISCIII, Madrid, Spain; 23 Universidad de Murcia, Murcia, Spain; 24 Fundación IDIBELL, Barcelona, Spain; 25 Universidad Autónoma de Madrid, Madrid, Spain; 26 Universidad Pompeu Fabra, Barcelona, Spain; 27 Agencia Estatal Consejo Superior de Investigaciones Científicas, Madrid, Spain; 28 Fundación IDIBELL, Barcelona, Spain; 29 Universidad de Zaragoza, Saragossa, Spain; 30 Hospital Clínico y Provincial de Barcelona, Barcelona, Spain; 31 Fundación Instituto de Investigación Sanitaria Illes Baleares (IdISBa), Palma, Spain; 32 Universidad Autónoma de Barcelona, Barcelona, Spain; 33 FPS/ELIXIR-ES, Andalusian Public Foundation Progress and Health-FPS, 41013 Seville, Spain

## Abstract

**Background:**

Despite being a very common type of genetic variation, the distribution of copy-number variations (CNVs) in the population is still poorly understood. The knowledge of the genetic variability, especially at the level of the local population, is a critical factor for distinguishing pathogenic from non-pathogenic variation in the discovery of new disease variants.

**Results:**

Here, we present the SPAnish Copy Number Alterations Collaborative Server (SPACNACS), which currently contains copy number variation profiles obtained from more than 400 genomes and exomes of unrelated Spanish individuals. By means of a collaborative crowdsourcing effort whole genome and whole exome sequencing data, produced by local genomic projects and for other purposes, is continuously collected. Once checked both, the Spanish ancestry and the lack of kinship with other individuals in the SPACNACS, the CNVs are inferred for these sequences and they are used to populate the database. A web interface allows querying the database with different filters that include ICD10 upper categories. This allows discarding samples from the disease under study and obtaining pseudo-control CNV profiles from the local population. We also show here additional studies on the local impact of CNVs in some phenotypes and on pharmacogenomic variants. SPACNACS can be accessed at: http://csvs.clinbioinfosspa.es/spacnacs/.

**Conclusion:**

SPACNACS facilitates disease gene discovery by providing detailed information of the local variability of the population and exemplifies how to reuse genomic data produced for other purposes to build a local reference database.

**Supplementary Information:**

The online version contains supplementary material available at 10.1186/s40246-023-00466-8.

## Background

Copy-number variations (CNVs) is a frequent form of genetic variation that is increasingly being linked to genetic and phenotypic diversity as well as to disease [1–3]. The interest in the assessment of CNVs is growing among the rare diseases community in recent years, given that they can explain cases that remain unsolved after conventional single nucleotide variant (SNV) prioritization [4]. Since there is a high level of natural (and apparently non-pathogenic) CNV mutational background [5, 6], it is important to have a reference repository that provides local population context and helps to distinguish these benign CNVs from potential pathogenic CNV findings in patients. As in the case of SNV variation, the local component of CNV variation is of utmost importance [7]. However, general databases, such as DECIPHER [8] or Gnomad [9] do not contain specific data from local populations that reflect the peculiarities of their CNV variant distributions. To offer this relevant aspect to genetic studies, the SPAnish Copy Number Alteration Collaborative Server (SPACNACS) stores CNV variation for more than 400 unrelated individuals of estimated Spanish ancestry. This database has been generated as a collaborative effort of the Spanish Network for Research in Rare Diseases (CIBERER) [10], the Navarra genome Project (NaGen) [11], and other research groups under a crowdsourcing cooperative model. Actually, our participation in projects like the undiagnosed patients programme (EnoD from the CIBERER) [12] guarantees a continuous submission of new patients to the database.

Since obtaining genomic data on a significant number of confirmed healthy people is often difficult, the strategy used here lies on the use of two annotations for the individuals: top levels of ICD10 and Human Phenotype Ontology (HPO) [13] annotations. This information allows building ad hoc queries in which the features of the studied individual are absent. In this way pseudo-control cohorts can be easily constructed in which, for example, Fanconi Anemia patients can take the role of pseudo-healthy reference for patients with retinal dystrophy (and vice versa). Although pleiotropies cannot be completely ruled out, they are expected to be infrequent across ICD10 top categories or high-level HPO terms.

The availability of the local population variability at the level of CNV opens the door to additional relevant studies, such as the contribution of natural copy number variation to the pharmacogenetic profile of the Spanish population. Because of the increasing abundance of genomic data, most of the genomic variations associated with pharmacogenomics are Single Nucleotide Variants (SNVs) and small indels [14], and the pharmacogenetic profile in the Spanish population has recently been described by us [15]. However, the role of CNVs in pharmacogenomic variation remains largely unknown and cannot be ignored [16]. A clear example of the potential role of these variants is the effect of the CNVs in the *CYP2D6* gene, which encodes an enzyme which is key in the metabolism of xenobiotics, including several drugs such as opioids [17], tricyclic antidepressants [18], selective serotonin reuptake Inhibitors [19], tamoxifen [20] or ondansetron/tropisetron [21].

The SPACNACS database is an example for future federated European infrastructures [22], whose aim is to enable discovery and analysis of genomic data without having direct access to them [23]. Moreover, SPACNACS is actively participating in the CNV specifications for the new Beacon 2.0 [24] standard that will facilitate the federated analysis of genomic data at CNV level for the first time. Interestingly, SPACNACS combines the discoverability possibilities offered by a Beacon with the possibility of contacting the group that generated the sequence, a useful feature present in tools like Matchmaker Exchange [25].

## Implementation

### Data

A subset of high-quality genomes and exomes from the Collaborative Spanish Variant Server database (CSVS) [15] were used to populate SPACNACS, and guarantee a continuous flux of new CNV data as CSVS grows. Genomic sequences come from different projects such as the Medical Genome Project [7, 26], the EnoD, (Undiagnosed Rare Diseases programme) from the Spanish Network for Research in Rare Diseases (CIBERER), the Project Genome 1000 Navarra, The RareGenomics [27] from Madrid, and other research groups and initiatives across Spain [28–30].

### Sample locality and potential kinship

The database contains only CNVs derived from unrelated individuals of Spanish ancestry. A leave-one-out cross-validation (LOOCV) strategy, previously used as a quality assessment to populate the CSVS [15], was utilized to build a distribution of percentages of variants contributed by any single sample to the pool of variants present in the rest of the database. Samples 1.5 times under the first interquartile range were considered genetically too close [15] and not included in the database. On the other hand, a Machine Learning based model, trained with different populations from the 1000 genomes project [31], was used to discriminate Spanish samples from the rest of populations (see [15] for details).

### Copy number variation predictions

For each sample, FASTQC v0.11.8 [32] was used to assess quality of raw data and fastp v0.20.0 [33] was run for quality preprocessing so that clean data is provided to downstream analysis. Then, filtered sequence reads were aligned to the reference human genome build hs37d5 (hg19) by using the BWA v0.7.16a alignment tool [34]. The obtained mapped reads (BAM files) were sorted by samtools v1.11 [35] and duplicated reads were marked to mitigate biases introduced by data generation steps by means of Picard tools v2.17.3 [36]. BAM files were later analyzed in terms of QC using in-house scripts and the ngsCAT v0.1 tool [37].

Two pipelines for predicting deletions and duplications were developed. One based on Manta v1.5.0 [38] and another on Gridss v2.7.3 [39]. In both cases, the best practices recommended in the documentation were followed. Only PASS variants were kept. Both predictions are available in SPACNACS and can be selected in the Search and Selection panel (see SPACNACS functionality section below).

### Pharmacogenomic analysis of the Spanish population

In order to evaluate the pharmacogenomic impact of CNVs in the Spanish population, we studied the variability of 1049 pharmacogenes involved in drug pharmacokinetics and/or drug response (Additional file 2: Table S2) described in PharmGKB database [14, 40]. We used Bedtools [41] and Pandas [42] to calculate the frequency of genes that overlap (totally or partially) with a deletion or a duplication in the Spanish population.

### Gene-phenotype associations

Gene-phenotypes associations were downloaded from the Human Phenotype Ontology database [13]. Primary HPO terms for individuals were manually assigned by clinicians and experts from the corresponding genomic projects mentioned above. Statistics and plots were generated with numpy [43], Pandas [42] and matplotlib [44] libraries.

### CNV annotations

SPACNACS includes an extensive annotation of CNVs with clinically relevant databases. A CNV is annotated with the features corresponding to the specific genomic region that overlaps. Such features include: (i) Clinvar database [45], a freely accessible, public archive of reports of the relationships among human variations and phenotypes, with supporting evidence. It also provides gene-disease relationships. (ii) DisGeNET [46], an exhaustive catalog of genes and variants associated with human diseases. DisGeNET integrates data from expert curated repositories, GWAS catalogs, animal models and the scientific literature. (iii) Gene Ontology Annotation (GOA) [47, 48], which contains a mixture of manual annotation and computationally assigned GO terms describing gene products. (iv) ClinGen [49], a National Institutes of Health (NIH)-funded resource that defines the clinical relevance of genes and variants for use in precision medicine and research. (v) The Human Phenotype Ontology [13], which provides a standardized vocabulary of phenotypic abnormalities encountered in human disease. In addition, HPO annotations derived from gene-phenotype links obtained from the analysis of a patient network [50]. (vi) The wgEncodeEH000322 track from UCSC [51], which provides information about the mappability of the genome. This annotation can be useful to detect false positives and biases in CNV prediction technologies/tools [52].

### Statistical methods

To estimate the functional enrichment in the CNV variants, the frequency of genes that overlap (totally or partially) with a deletion or a duplication in the Gridss pipeline prediction dataset was first calculated. Genes affected by more than one CNV of the same individual were only counted once. Then, a z-score normalization of the frequency was performed, and those genes with a score greater or equal to four were selected. Finally, the web tool metascape [53] was used to carry out the functional enrichment.

## Results

### SPACNACS content description

**Fig. 1:**
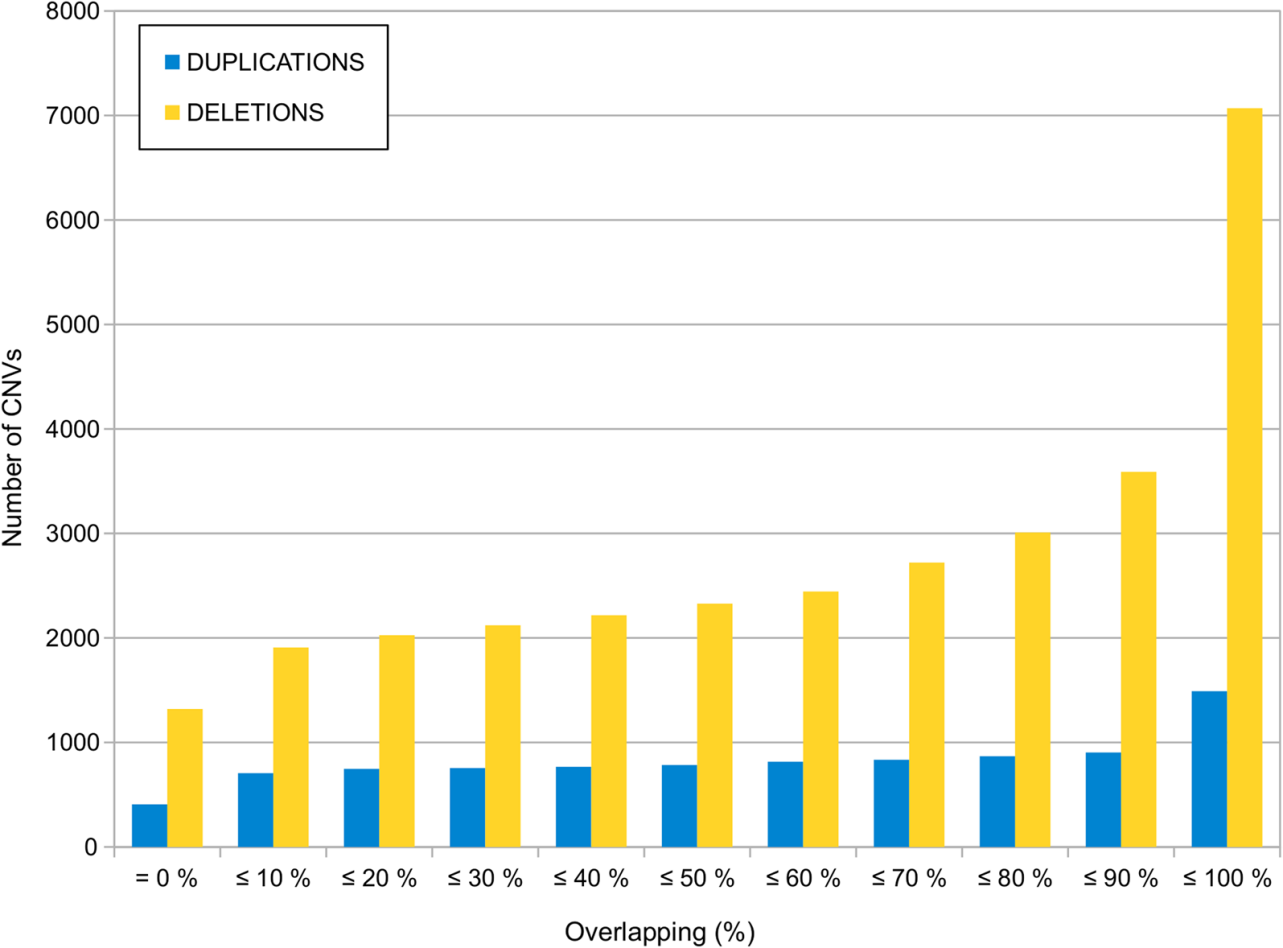
CNVs overlapping between SPACNACS and other databases. Comparative between the CNVs found in SPACNACS and the ones present in the 1000 genomes and Gnomad databases. The *X* axis incrementally represents the level of overlap between the CNVs compared, which range from 0 (CNVs unique to SPACNACS) up to 100% (CNVS with a perfect match)

Focusing on the Gridss pipeline, SPACNACS contains a total of 8559 unique CNVs, corresponding to 7069 deletions (83%) and 1490 duplications (17%). The reciprocal overlapping between these CNVs and those contained in two other population databases (1000 genomes project and Gnomad [9]) was evaluated. As a result, most SPACNACS CNVs overlap to a greater or lesser extent with some CNVs from both, 1000 genomes or Gnomad (Fig. 1). A total of 586 duplications (39% of the total duplications) and 3479 deletions (49% of the total deletions) of SPACNACS match almost completely with CNVs present in 1000 genomes and/or Gnomad (overlapping greater than 90%, which corresponds to the increment between the penultimate and the ultimate bars in Fig. 1). Interestingly, a remarkable amount of SPACNACS CNVs do not overlap with any other CNV from 1000 genomes or Gnomad (see the first column of bars in Fig. 1), specifically 407 duplications (27% of the total duplications) and 1320 deletions (19% of the total deletions). These CNVs can be considered a priori either exclusive of the Spanish population or, at least, CNVs which are very abundant in the Spanish population but scarce in other populations. Therefore, these CNVs would play a crucial role in CNVs prioritization processes. The rest of CNVs display a partial overlap with CNVs present in the 1000 genomes and Gnomad databases.

From the analysis of 417 individual samples (processed with the Gridss pipeline), a total of 26,623 different sequences, corresponding to diverse biotypes affected by at least one CNV, were observed. Among them, 10,347 sequences (40%) are protein coding genes (see Additional file 1: Table S1), while the rest correspond to other biological categories (pseudogenes, RNAs, etc.). As expected, most of these genes are rarely affected by CNVs in the SPACNACS sample of the local population. Only 1064 (10.2%) coding genes are affected by at least one CNV in more than 5% of the individuals.

Since SPACNACS is composed of patients of different diseases (binned in subpopulations corresponding to the highest ICD10 levels) it is expectable that CNVs shared by several subpopulations will not be disease-specific. Given that some subpopulations can have some overlap (e.g. “5: *Mental and behavioural disorders”*, “6: *Diseases of the nervous system”*, “17: *Congenital malformations, deformations and chromosomal abnormalities”*, and the corresponding controls “31: *Mental and behavioural disorders—controls”*, “32: *Diseases of the nervous system—controls”*, “43: *Congenital malformations, deformations and chromosomal abnormalities—controls”*), a threshold of 15 different subpopulation affected have been chosen to ensure that CNVs are not disease-specific. There are 771 genes contained in CNVs that appear in individuals belonging to a total of 15 or more subpopulations. These genes constitute a conservative estimation of the genes affected by CNVs with likely no pathogenic consequences. Interestingly, 189 of them have an OMIM annotation corresponding to different inherited diseases (see Additional file 1: Table S1). This suggests that, while point mutations affecting the functionality of the gene have a pathologic effect, dose effects due to copy number alterations of the whole genes are unlikely to imply pathogenicity. Generally speaking, genes of recessive disorders would not produce any phenotype in a deletion, providing a chromosome copy is preserved. It is also likely that a proper transcriptional control can cope with deficiencies or overabundances in the number of genes [54, 55].

Among the most frequently affected genes, there is a significant (*p*-value = 2.34*10^–6^) functional enrichment, according to the metascape tool [53] (see statistical methods section), in genes involved in *detection of chemical stimulus involved in sensory perception* (GO:0050907). This observation is in line with the fact that most olfactory receptors genes are located in segmentally duplicated regions, which are known to be frequently involved in regions affected by copy-number variation [56, 57]. Other biological functions detected in the enrichment are of more complex interpretation, given that are very general terms, such as *cell killing* (GO:0001906; *p*-value = 0.00065), *single fertilization* (GO:0007338, *p*-value = 0.0026), *anatomical structure maturation* (GO:0071695, *p*-value = 0.0068) and *neuron projection morphogenesis* (GO:0048812, *p*-value = 0.0093).

### SPACNACS architecture

The SPACNACS is a web server that can be found at: http://csvs.clinbioinfosspa.es/spacnacs/. The front-end has been developed using the JavaScript REACT library v17.0.2 [58]. The genome browser has been built using the IGV.js library [59]. The Integrative Genomics Viewer (IGV) is a popular high-performance, easy-to-use, interactive tool for the visual exploration of genomic data [60]. The back-end has been written in Java programming language and the database has been built using Mongo [61], a NoSQL document database used to build highly available and scalable internet applications.

### SPACNACS functionality

**Fig. 2:**
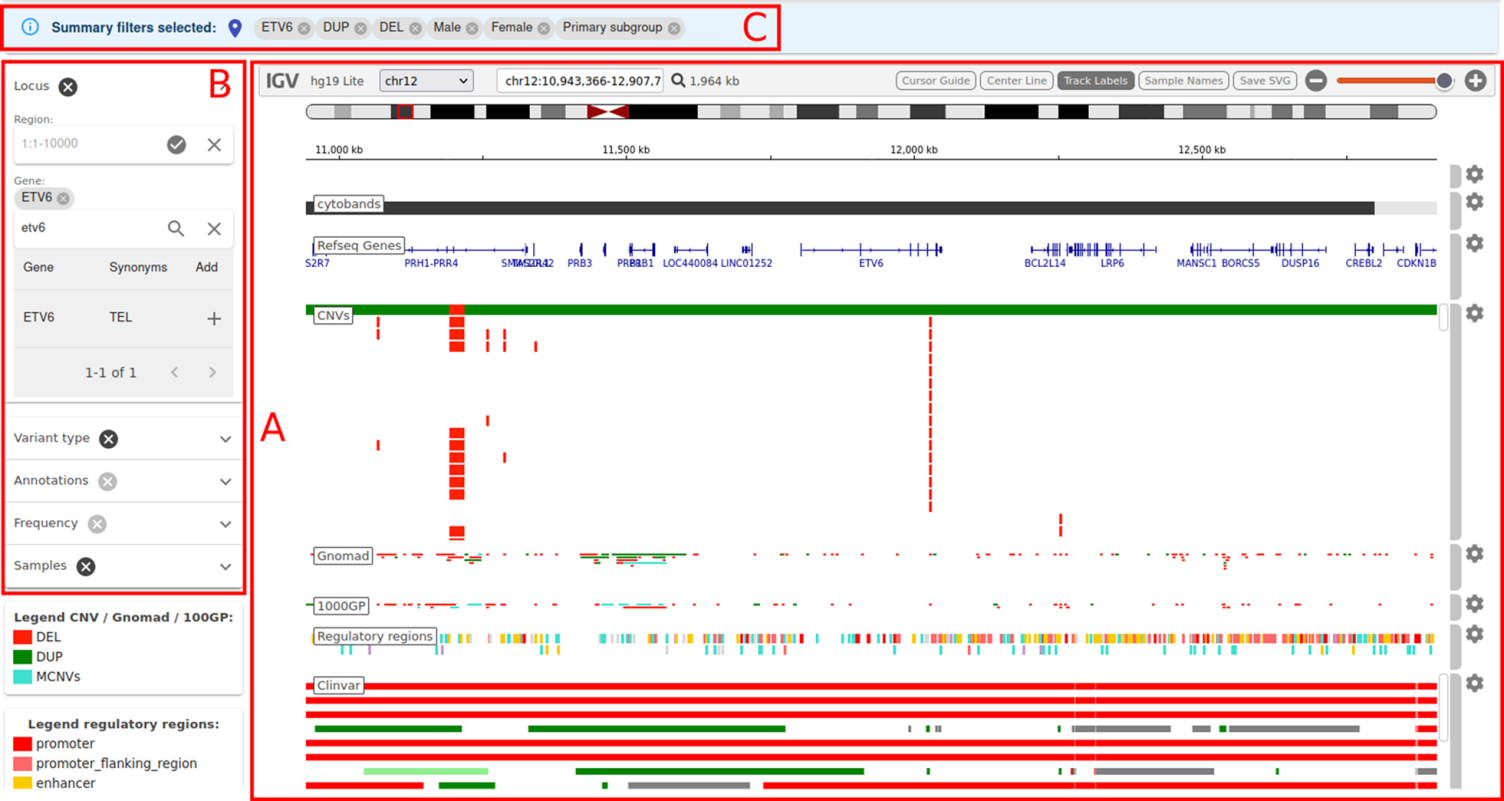
The SPACNACS interface. **A** Genome browser panel consisting of an embedded Integrative Genomics Viewer preloaded with the Spanish CNV database and other useful tracks. **B** Search and selection panel, which provides several filters for specifying the genomic region and the data to be shown. **C** Filtering status panel, which shows information about the active filters. The whole dataset is shown by default

The web interface has 3 main sections: (i) The Browser panel (Fig. 2A), which consists of an embedded Integrative Genomics Viewer [60] preloaded with the Spanish CNV database, world population CNVs derived from Gnomad v2.1 control samples [62] and the 1000 Genomes Project structural variants phase 3 [63], regulatory regions as provided by Ensembl [64] and known clinically relevant copy number gains, copy number losses, duplications and deletions from ClinVar [45]. The browser provides several navigation controls for specifying the genomic region to view. (ii) Search and selection panel (Fig. 2B). This section controls which CNVs are displayed in the CNVs track. Several filters for specifying the genomic region and the data to be shown are provided. For example, filtering by *intellectual disability* (C3714756) term in DisGeNET will show only CNVs overlapping genes associated with this phenotype in DisGeNET. In a similar way, selecting a subset of samples according to the ICD10 category, gender or main HPO term will automatically update the CNV frequency. This allows researchers to use this repository as a pseudo-control population for ruling out non-causal CNVs and helping to find new disease-causing CNVs. In the selection panel the type of pipeline used to infer the CNVs, Manta [38] or Gridss [39] (see Methods) can be selected. (iii) Filtering status panel (Fig. 2C), which shows information about the active filters. By default, the whole dataset is selected. Additional information about the interface can be found at the server documentation main page (https://github.com/babelomics/SPACNACS/wiki).

### The SPACNACS beacon

SPACNACS implements a Beacon (version 1.0), a standard protocol used to query the database to check whether a specific region is involved in a CNV. The Beacon is an initiative of the Global Alliance for Genomics & Health (GA4GH) that allows genomic data sharing across federated networks [65]. The Beacon is a web-accessible service that can be queried for information about one specific allele at a time. For example, in the classical Beacon a user can pose queries of the form “Have you observed this particular variation (e.g., nucleotide A) at this genomic location (e.g., position 21,926,123 on chromosome 8)?” to which the Beacon responds with either “yes” or “no.” Here, the Beacon allows queries on amplifications or deletions that involve regions. Since the definition of the boundaries of the CNVs is often difficult, the Beacon allows querying with ranges. The generic URL to query the SPACNACS beacon is as follows:http://csvs.clinbioinfosspa.es:8080/spacnacs-ga4gh-beacon-v1/query?referenceName=[chromosome]&referenceBases=N&assemblyId=GRCh37&startMin=[starMin]&startMax=[starMax]&endMin=[endMin]&endMax=[endMax]&variantType=[DUP/DEL].

The Beacon 1.0 is used to query a region containing any base (reference Bases = *N*). For example, to search for any CNV (the & variant Type parameter is dropped) in the chromosome 8, in the locus spanning between positions 22,138,284 and 22,138,339 DEL within an interval of ± 10 nucleotides the query would be as follows:http://csvs.clinbioinfosspa.es:8080/spacnacs-ga4gh-beacon-v1/query?referenceName=8&referenceBases=N&assemblyId=GRCh37&startMin=22138273&startMax=22138293&endMin=22138328&endMax=22138348.

SPACNACS is participating in the definition of Beacon specifications for CNV-related queries in the new Beacon 2.0 standard, currently under development [24].

### Impact of CNV in human phenotype

Individuals in SPACNACS come from CSVS with detailed HPO annotations made by experts from the different genomic projects of origin. This allows carrying out an interesting correlation between phenotype and genes with phenotypic annotations in the different diseases present in the database. Table 1 lists the different HPOs found in individuals present in the database along with the HPO-related genes affected by CNVs. There are phenotypes, like *Intellectual disability*, *Global developmental delay*, *Ataxia*, *Microcephaly*, and nine more, in which all the CNVs present in affected individuals overlap genes annotated with HPOs corresponding to the phenotype. It is important to note that it does not mean that the causal genetic variation of the disease in all these individuals is a CNV. Actually, it could be the case that the diagnosis was due to a SNV for some individuals. However, it is remarkable that in some cases all the CNVs overlap, and presumably affect, genes with HPOs corresponding to the phenotype of the individual. This suggests an important role of copy number variation in some phenotypes. Actually, it is well known the role of structural variation in intellectual disabilities [66]. In other cases, like breast carcinoma, only in 10% of the patients a gene related to this HPO was affected by a deletion. In a wider spectrum of HPOs, none of the HPO-related genes was affected by a CNV. Several cancers, cardiomyopathies and retinopathies are examples of diseases typically caused by SNVs and only in an infrequent number of cases by CNVs, which agrees with the observations summarized in Table 1.

**Table 1:** HPOs in the individuals and HPO-related genes affected by CNVs in them

HPO ID	HPO	Individuals	Deletions	Amplifications	Any CNV	Percentage explained
HP:0001249	Intellectual disability	17	17	7	17	100.00
HP:0001263	Global developmental delay	17	17	10	17	100.00
HP:0001251	Ataxia	5	5	0	5	100.00
HP:0000252	Microcephaly	4	4	3	4	100.00
HP:0004322	Short stature	4	4	0	4	100.00
HP:0001298	Encephalopathy	3	3	0	3	100.00
HP:0002652	Skeletal dysplasia	2	2	0	2	100.00
HP:0001256	Intellectual disability, mild	1	1	0	1	100.00
HP:0001328	Specific learning disability	1	1	0	1	100.00
HP:0001270	Motor delay	1	1	0	1	100.00
HP:0001250	Seizures	1	1	0	1	100.00
HP:0001290	Generalized hypotonia	1	1	1	1	100.00
HP:0001067	Neurofibromas	1	1	0	1	100.00
HP:0200134	Epileptic encephalopathy	4	3	0	3	75.00
HP:0000729	Autistic behavior	2	1	0	1	50.00
HP:0001332	Dystonia	3	1	0	1	33.33
HP:0010864	Intellectual disability, severe	6	1	0	1	16.67
HP:0003002	Breast carcinoma	10	1	0	1	10.00
HP:0000083	Renal insufficiency	9	0	0	0	0.00
HP:0002206	Pulmonary fibrosis	6	0	0	0	0.00
HP:0000107	Renal cyst	5	0	0	0	0.00
HP:0002313	Spastic paraparesis	5	0	0	0	0.00
HP:0003003	Colon cancer	5	0	0	0	0.00
HP:0000488	Retinopathy	4	0	0	0	0.00
HP:0002664	Neoplasm	3	0	0	0	0.00
HP:0002110	Bronchiectasis	3	0	0	0	0.00
HP:0002342	Intellectual disability, moderate	2	0	0	0	0.00
HP:0011343	Moderate global developmental delay	2	0	0	0	0.00
HP:0009830	Peripheral neuropathy	2	0	0	0	0.00
HP:0001300	Parkinsonism	2	0	0	0	0.00
HP:0001258	Spastic paraplegia	2	0	0	0	0.00
HP:0012126	Stomach cancer	2	0	0	0	0.00
HP:0001638	Cardiomyopathy	2	0	0	0	0.00
HP:0003107	Abnormal circulating cholesterol concentration	2	0	0	0	0.00
HP:0004482	Relative macrocephaly	1	0	0	0	0.00
HP:0000256	Macrocephaly	1	0	0	0	0.00
HP:0008551	Microtia	1	0	0	0	0.00
HP:0000525	Abnormality iris morphology	1	0	0	0	0.00
HP:0007105	Infantile encephalopathy	1	0	0	0	0.00
HP:0002134	Abnormality of the basal ganglia	1	0	0	0	0.00
HP:0007002	Motor axonal neuropathy	1	0	0	0	0.00
HP:0003477	Peripheral axonal neuropathy	1	0	0	0	0.00
HP:0003198	Myopathy	1	0	0	0	0.00
HP:0001324	Muscle weakness	1	0	0	0	0.00
HP:0010978	Abnormality of immune system physiology	1	0	0	0	0.00
HP:0100242	Sarcoma	1	0	0	0	0.00
HP:0008527	Congenital sensorineural hearing impairment	1	0	0	0	0.00
HP:0008504	Moderate sensorineural hearing impairment	1	0	0	0	0.00
HP:0001639	Hypertrophic cardiomyopathy	1	0	0	0	0.00
HP:0004356	Abnormality of lysosomal metabolism	1	0	0	0	0.00
HP:0032245	Abnormal metabolism	1	0	0	0	0.00

### Impact of CNVs on genes relevant in pharmacogenomics

**Fig. 3:**
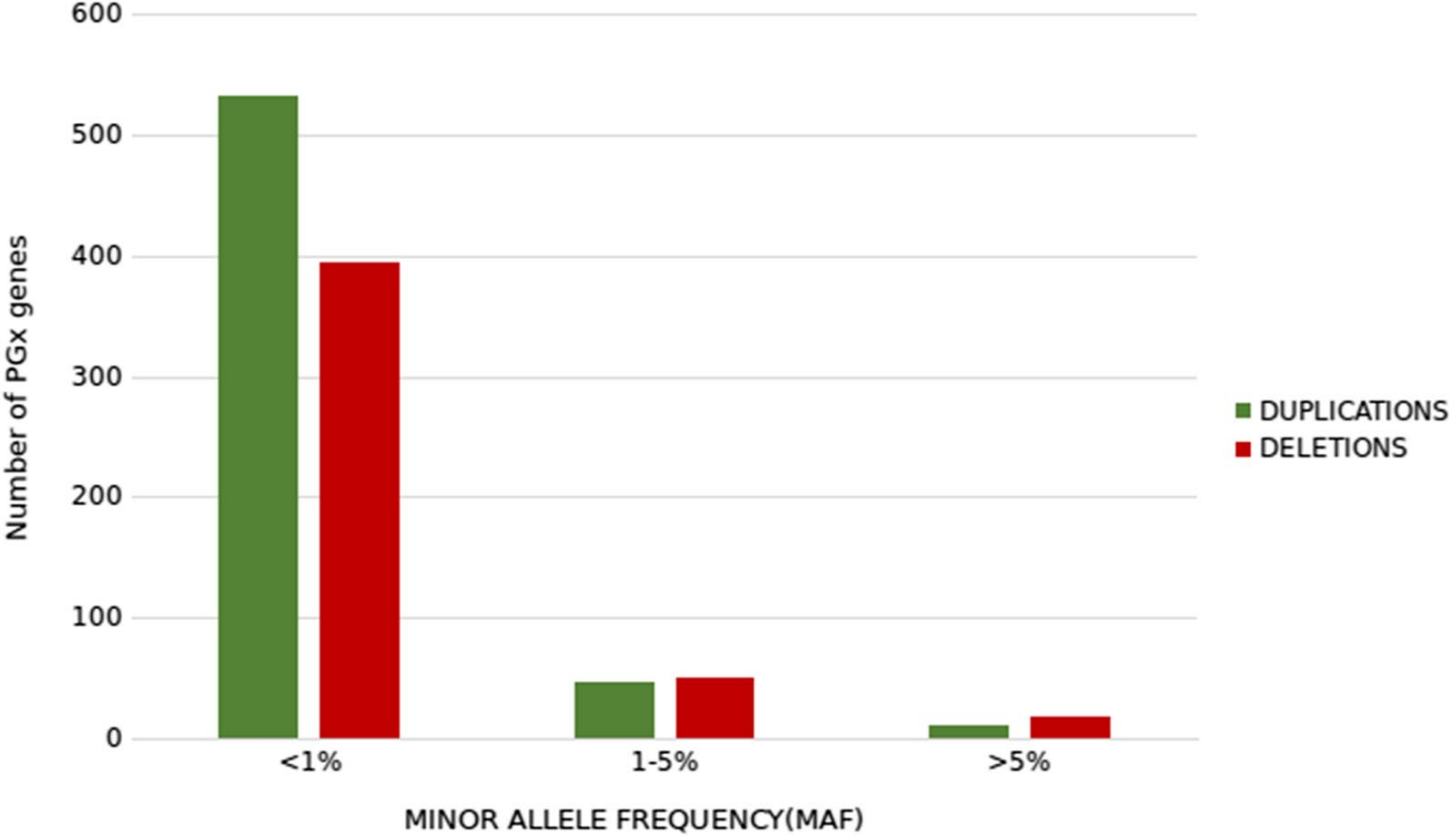
Distribution of CNVs detected in pharmacogenes (PGx genes) according to the allele frequency. The number of genes harboring duplications (green) or deletions (red) of the 1045 PGx genes tested are shown according to the frequency detected in the population

In order to know the contribution of CNVs to the variability of pharmacogenomic relevant genes, the number of copies of a total of 1049 genes, reported in the clinical annotations from PharmGKB database [14], was assessed. Almost three quarters of these genes (71.7%, 749 out of 1045 pharmacogenomics genes) were involved in CNVs, with almost a half of them (49.6%, 518 out of 1045) with the full length of the gene affected by a CNV. These results document a non-negligible potential impact of CNVs on pharmacogenetic protein function. Duplications were found in a slightly more frequent proportion (56.03%) than deletions (43.97%) (Fig. 3), with 29.1% of the genes harboring both kinds of events. Of note, a 5.59% of the genes showed a minor allele frequency (MAF) higher than 5% in the Spanish population suggesting a relevant role of this kind of variation in the pharmacogenomics field. More interestingly, when the analysis was carried out only for the 21 well-described ‘actionable’ pharmacogenomics genes (PharmGKB level 1A), 80.95% of them showed CNVs with aggregated frequency of 8.29%, excluding *CYP2D6* contribution. The *CYP2D6* analysis revealed a lower CNVs frequency in the Spanish population compared with the data reported for Caucasians (< 1% versus 5.7%) [17]. These differences could be explained because the algorithm used for CNV detection is not able to solve the complexity of the structural variant rearrangements between *CYP2D6* and the pseudogene *CYP2D7* next to the gene [67–69].

## Discussion

CNVs are a pervasive form of genetic variation and, as more data are collected, they are increasingly being linked to phenotypic diversity and disease [1–3, 70]. Particularly, somatic CNVs are observed in the majority of cancer types and are known to have a clear impact in cancer development and progression [71, 72]. A comprehensive representation of somatic genomic variation profiles in cancer can be found in the Progenetix database [73]. Also, numerous reports support a significant role of germinal CNVs in neurodevelopmental disorders and multiple congenital abnormalities [74, 75]. For example, more than 12% of neurodevelopmental disorder cases can be explained by a CNV [66]. It has been reported that up to 15% of the autism spectrum could be explained by CNVs that are either de novo or rarely inherited in nature [76, 77]. Because the most characterized penetrant CNVs are inherently rare, comparative analyses against reference populations are required to assess relative disease risk and to elucidate the potential etiologic role of such genetic events, currently classified as “variants of unknown significance” (VUS) [66]. An important step in the discovery of genomic variation causal of diseases is the detailed knowledge of the local variability, as has been highlighted in the case of SNPs, where many databases with local variability have appeared in the last years [15, 78–82]. Since CNV data have traditionally been obtained at a much slower pace than other omic data, such as whole genome or whole exome sequences or transcriptomic data, finding these CNV reference population databases is often difficult. The necessity of addressing a wide range of CNV-related challenges ranging from detection and interpretation, including the lack of CNV databases with reference populations with a local component has been identified by the ELIXIR’s recently established human CNV Community [23]. Here, SPACNACS has followed the philosophy of CSVS [15], which has been described as an especially interesting example of how to collect and distribute genomic data [83], and has built a local reference of the CNV variation in the Spanish population by collecting data from different genomic projects. Another interesting feature of SPACNACS is that, instead of trying to use different tools to infer a consensus CNV profile, here, two different approaches, Manta (52) and Gridss (53) have been used and are available. Since there is not a consensus pipeline for the detection of CNVs it is interesting that people using different pipelines can find in SPACNACS different CNV estimations.

As a demonstration of the usefulness of this resource two studies using SPACNACS data are presented here. Firstly, a study of the degree of matching between the HPO annotation of individuals in SPACNACS and the corresponding HPO-related genes affected by CNVs found in them. Interestingly, the higher affectation of HPO-related genes by CNVs occurs in HPOs corresponding to diseases in which structural variation plays an important role in the etiology, such as mental disabilities or related developmental malformations [66].

Another interesting aspect is the imbalance between CNV types: deletions are significantly more frequent than duplications, a disproportion which has also been observed in other databases, such as Gnomad [9]. Actually, this imbalance is long known, as it was described that 29% of genetic diseases were caused by CNVs, being 22% of the deletions and only 7% duplications [84]. Moreover, another study in which spontaneous duplication and deletion rates were compared to observed CNV polymorphism data from sequenced genomes, suggest that the most gene duplications are likely detrimental and are removed by natural selection [85], which can explain the observed imbalance between deletions and duplications. However, this trend is not observed in pharmacogenomic genes, which are affected almost equally by duplications (56.03%) and deletions (43.97%). Speculating the reasons for this observation is beyond the scope of this manuscript. It could be due to the fact that partial loss of function in pharmacogenomic genes is similar in deletions and duplications, contrarily to the case of essential genes mentioned above, causative of genetic diseases. Alternatively, it might be simply a matter of sampling, because the number of genes pharmacogenomic genes is not high.

Also, in spite of some limitations due to the complexity of pharmacogenomics variation, SPACNACS has demonstrated to be a valuable tool for exploring CNVs contribution in this type of genomic alteration, providing primary data about reference frequencies of pharmacogenomic genes in the Spanish population. Thus, the data presented here points to CNVs as a relevant type of variation for pharmacogenomic diagnosis and suggests their use, along with that of SVNs in the clinical implementation of pharmacogenomics.

## Supplementary Information

**Additional file 1: Table S1.** List and annotation of protein coding genes affected by at least one CNV in SPACNACS samples processed with Gridss pipeline.

**Additional file 2: Table S2.** List of genes involved in drug pharmacokinetics and/or drug response.

## Data Availability

Project name: SPACNACS. Project home page: http://csvs.clinbioinfosspa.es/spacnacs. Operating system: Platform independent. Programming language: Javascript, Java and MONGO database. License: freely accessible web server.

## Abbreviations

CNV: Copy number variation
HPO: Human phenotype ontology
ICD10: International classification of diseases version 10
MAF: Minor allele frequency
VUS: Variants of unknown significance
